# US Centers for Disease Control and Prevention and Its Partners’ Contributions to Global Health Security

**DOI:** 10.3201/eid2313.170946

**Published:** 2017-12

**Authors:** Jordan W. Tappero, Cynthia H. Cassell, Rebecca E. Bunnell, Frederick J. Angulo, Allen Craig, Nicki Pesik, Benjamin A. Dahl, Kashef Ijaz, Hamid Jafari, Rebecca Martin, Global Health Security Science Group

**Affiliations:** Centers for Disease Control and Prevention, Atlanta, Georgia, USA

**Keywords:** global health security, global health protection, public health, emergency response, International Health Regulations, Centers for Disease Control and Prevention, Field Epidemiology Training Program, vaccine-preventable diseases

## Abstract

To achieve compliance with the revised World Health Organization International Health Regulations (IHR 2005), countries must be able to rapidly prevent, detect, and respond to public health threats. Most nations, however, remain unprepared to manage and control complex health emergencies, whether due to natural disasters, emerging infectious disease outbreaks, or the inadvertent or intentional release of highly pathogenic organisms. The US Centers for Disease Control and Prevention (CDC) works with countries and partners to build and strengthen global health security preparedness so they can quickly respond to public health crises. This report highlights selected CDC global health protection platform accomplishments that help mitigate global health threats and build core, cross-cutting capacity to identify and contain disease outbreaks at their source. CDC contributions support country efforts to achieve IHR 2005 compliance, contribute to the international framework for countering infectious disease crises, and enhance health security for Americans and populations around the world.

To contain health threats and ensure global health security, all countries must rapidly detect and respond to public health emergencies and, when overwhelmed, call upon global deployment capacity. This need is clearly evident, as the world is more susceptible to infectious disease threats due to increased international travel and trade, spread of newly emerging or reemerging microbes, and inadvertent release of dangerous pathogens from laboratories or bioterrorism acts.

Following the 2002–2003 severe acute respiratory syndrome (SARS) coronavirus outbreak, which demonstrated how rapidly a pathogen could spread to 26 countries (*1*), the World Health Organization (WHO) in 2005 adopted the revised International Health Regulations (IHR 2005), a legally binding international treaty. In June 2007, all 196 WHO member states committed to reaching IHR 2005 compliance by 2012 (*2*). The 2009 pandemic of influenza A(H1N1) resulted in the first declaration of a public health emergency of international concern under IHR 2005 (*3*) and provided new evidence that the world was ill prepared for a global health crisis. Numerous threats followed H1N1, including cholera in post-earthquake Haiti in 2010 (*4*); Middle East respiratory syndrome coronavirus in Saudi Arabia in 2012 (*5*) and its exportation to the Middle East, Europe, Asia, and the United States; West Africa Ebola virus disease in 2014 (*6*); chikungunya virus in 2013 and Zika virus in 2015 in the Americas (*7*); and yellow fever virus reemergence in Africa, China, and Brazil in 2015 (*8*). Despite these serious threats, only 33% of WHO member states had self-reported IHR 2005 compliance by December 2014 (*9*).

Building and maintaining global preparedness for pandemic threats and IHR 2005 compliance requires coordination and technical expertise across multiple stakeholders. To protect Americans and the global community from health threats, the US Centers for Disease Control and Prevention (CDC) has established a global health protection platform that works with ministries of health (MOHs); other partners (e.g., host country partners, WHO, nongovernmental organizations, and academic institutions); CDC country offices; and agency programs, including those dealing with influenza, emerging zoonotic diseases, HIV, malaria, and polio (*10*). CDC has also worked on building cross-cutting core capacities to ensure protection from these specific diseases and unpredictable new health threats through initiatives such as the Field Epidemiology Training Program (FETP) and the Global Disease Detection (GDD) network (*11**,**12*). This report highlights selected CDC global health protection platform accomplishments, enhanced through the Global Health Security Agenda (GHSA), that strengthen emergency mitigation and capacity-building partnerships dedicated to containing threats at their sources.

## Emergency Mitigation of Global Health Threats

### Ending the West Africa Ebola Outbreak

The unprecedented 2014–2016 West Africa Ebola epidemic devastated Guinea, Liberia, and Sierra Leone, 3 of the world’s poorest nations (*13*). These countries accounted for >99% of reported cases (28,652) and deaths (11,325) from Ebola virus infections (*6*). Ending the epidemic required enormous efforts from affected countries and collaborations with international partners, including CDC (*6*). CDC supported >3,500 staff deployments, engaging in epidemiologic fieldwork, laboratory testing, risk-reduction communications, improvements in infection control, and research on risk factors for transmission, viral persistence, and an Ebola vaccine (Table 1).

**Table 1:** Selected US CDC global health protection platform accomplishments*

Global health protection accomplishments	Number	Timeframe
Emergency mitigation of global health threats		
Ending the West Africa Ebola outbreak		
CDC staff deployments overall, domestic and international	>3,500	2014–2016
Departing passengers in the 3 affected countries screened for Ebola virus disease	>339,000	2014–2016
Vaccinations of health workers in Ebola trial	>8,000	2015
Days of continuous operation of high-throughput laboratory capacity in Sierra Leone; >23,000 specimens tested	421	2014–2015
US healthcare workers trained in Anniston, AL, to work in West Africa	>600	2015
GRRT		
CDC-trained GRRT experts prepared to deploy on short notice to a public health emergency	>400	2017 (Jun)
GRRT mobilizations (>14,000 cumulative person-days), supporting responses to global health emergencies including Zika, yellow fever, cholera, measles, polio, and Ebola	>420	2015–2017 (Jun)
Rapid humanitarian responses		
Staff deployments in response to public health humanitarian emergencies in >40 countries	>380	2011–2016
Staff deployments to 6 countries in response to Syria crisis	85	2012–2016
Countries with morbidity/mortality surveillance systems implemented in response to Horn of Africa famine	3	2011–2012
PHEM program		
Fellows from 28 countries trained through CDC PHEM fellowship	69	2013–2017 (Jun)
Countries that have received CDC emergency management technical assistance and training	56	2013–2016
Countries that participated in a real and/or simulated response with CDC technical assistance	19	2013–2016
Global Disease Detection Operations Center		
Serious public health threats assessed	>1,500	2007–2016
Countries where serious outbreaks were investigated/contained, where CDC provided technical assistance	>190	2007–2016
Unique diseases tracked globally	>170	2007–2016
Outbreaks monitored and reported in >130 countries for ≈40 different diseases	≈300	2016
GDD activities		
GDD regional centers	10	2006–2016
New diagnostic tests established in national or regional laboratories	>380	2006–2016
New strains/pathogens detected and/or discovered (new to the world, new to country or region, or new modes of transmission likely because of increased ability to detect through newly introduced laboratory tests) in which GDD assisted in detection and identification	79	2006–2016
Outbreaks responded to by GDD center that provided epidemiology and/or laboratory assistance	2,051	2006–2016
Outbreak investigations in which laboratory support was provided	1,363	2006–2016
Participants who received public health trainings conducted at national and/or regional level on topics, including epidemiology, laboratory, all-hazards preparedness, and risk communication	115,566	2006–2016
Capacity-building partnerships to contain threats at the source		
GHSA implementation		
GHSA countries: 17 Phase I countries, 14 Phase II countries, and CARICOM†	>31	2015–2017 (Mar)
Phase I countries with enhanced surveillance systems for zoonotic diseases	13	2015–2017 (Mar)
Countries that detected dangerous pathogens using new equipment and capabilities	16	2015–2017 (Mar)
Phase I countries supported in development of Emergency Operations Centers	16	2015–2017 (Mar)
Joint External Evaluation		
GHSA assessments conducted before tool finalization	6	2016
Evaluations completed	52	2016–2017 (Jul)
Public health workforce development		
Countries with CDC-supported FETPs	65	1980–2016
Graduates of FETPs-Advanced	>3,900	1982–2016
Outbreaks investigated by FETPs-Advanced trainees	>3,300	2005–2016
New FETPs-Frontline started	24	2014–2016
Participants in FETPs-Frontline	>1,860	2015–2016
Global vaccine-preventable disease activities		
STOP program volunteers trained in surveillance principles to detect and respond to cases of polio and other vaccine-preventable diseases	2,010	1998–2017 (Jul)
Countries with volunteers deployed for the STOP program	77	1998–2016
Countries supported by CDC to build national STOP programs	4	1998–2016
NPHIs		
Members of International Association of National Public Health Institutes and supported by CDC	>100	2016
Countries receiving NPHI development support from CDC	>20	2016
Persons across the globe served by NPHIs	5 billion	2016

**Figure 1:**
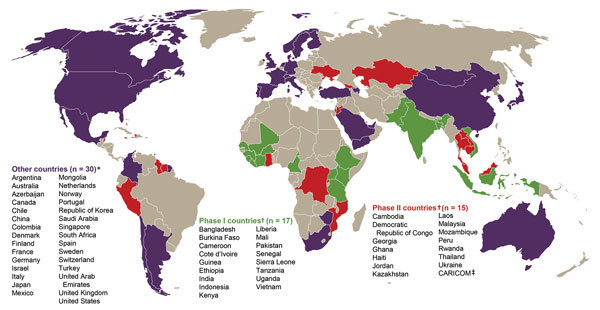
Global Health Security Agenda (GHSA) member countries as of July 25, 2017 (https://www.ghsagenda.org/members). *GHSA member countries that are not directly supported by the US government. †US government–supported GHSA member countries. CDC provides technical assistance to support country capacity assessments, the development of 5-year GHSA road maps, and annual GHSA implementation plans in Phase I, Phase II, and CARICOM nations. In the Phase I countries, CDC also provides financial support for implementation of the GHSA Action Packages. ‡CARICOM is an organization of 15 island nations. CARICOM, Caribbean Community; GHSA, Global Health Security Agenda.

*CARICOM, Caribbean Community; CDC, Centers for Disease Control and Prevention; FETP, field epidemiology training program; GDD, Global Disease Detection; GHSA, Global Health Security Agenda; GRRT, Global Rapid Response Team; NPHI, National Public Health Institute; PHEM, public health emergency management; STOP, Stop Transmission of Polio. †CARICOM is an organization of 15 Caribbean nations and dependencies. In 2015, the US government committed to accelerating GHSA implementation with 31 countries and CARICOM (Figure 1). In 17 Phase I, 14 Phase II, and CARICOM nations (Figure 1), CDC provides technical assistance to support country capacity assessments, the development of 5-year GHSA road maps, and annual GHSA implementation plans. In the Phase I countries, CDC also provides financial support for implementation of the GHSA action packages (Table 2) (*14**–**16*).

In December 2014, the US Congress authorized $1.2 billion in emergency funding for CDC to end the Ebola epidemic and accelerate GHSA implementation in partnering countries (Figure 1) (*14*). In early 2015, these funds made it possible for CDC to augment its response with new CDC country offices in Guinea, Liberia, and Sierra Leone, which enhanced response activities to end the epidemic. These countries are now implementing GHSA to build national resilience and preparedness capability (Table 1; Figure 1). Key examples of this work’s impact are the efficient identification and control of the past 7 Ebola virus clusters (*17*) during 2015–2016 and the rapid response to a cluster of deaths from *Neisseria meningitidis* infection in Liberia in 2017 (*18*). These countries are demonstrating that they are now better prepared to prevent, detect, and respond to serious disease threats (Table 2).

**Table 2:** Global Health Security Agenda’s prevent, detect, and respond framework against infectious disease threats and its 11 measurable action packages (*14**,**15*)

Steps and actions
Prevent: systems, policies, and procedures to mitigate avoidable outbreaks
Surveillance to guide slowing of antimicrobial resistance
National biosecurity system
Policies and practices that reduce the risk of zoonotic disease transmission
Immunization of 90% of children <1 year of age with >1 dose of measles vaccine
Detect: a national surveillance and laboratory system capable of reliable testing for >5 of 10 core tests relevant to the country’s epidemiologic profile on specimens from disease clusters in >80% of districts
Standardized surveillance for 3 core syndromes
Regional and national interoperable electronic reporting systems
Timely reporting to World Health Organization (WHO), World Organisation for Animal Health (OIE), and Food and Agriculture Organization of the United Nations (FAO)
Multidisciplinary public health workforce with ≥1 epidemiologist per 200,000 population
Respond: a national public health Emergency Operations Center capable of activating an emergency response in <2 hours
Trained rapid response teams
Linkages between public health and law enforcement for suspected biologic attacks
National framework to engage international partners during a public health emergency

### Global Rapid Response Team

The 2014–2016 West Africa Ebola epidemic was the largest emergency response in CDC’s history (*6*). The identification, training, and deployments of >3,500 CDC staff taxed agency human resource systems and challenged response continuity in the early months. To ensure sustained readiness for the next health emergency, CDC now trains and rosters a Global Rapid Response Team of >400 experts with a broad range of technical and language skills, poised to deploy on short notice and remain in the field for up to 6 months (Table 1).

During September 2015–June 2017, these responders were mobilized >420 times and contributed >14,000 cumulative person-days to emergency response in the field, in Atlanta’s Emergency Operations Center (EOC), or both (Table 1). During this period, the Global Rapid Response Team responded to 13 emergencies in 25 countries, including Zika virus in the Americas (217 mobilizations, 9,494 person-days, 15 countries and territories, and EOC); yellow fever in Angola and the Democratic Republic of the Congo (20 mobilizations and 1,097 person-days); Hurricane Matthew in Haiti (59 mobilizations and 1,235 person-days); and, most recently, Ebola virus in the Democratic Republic of the Congo.

### Rapid Humanitarian Responses

Humanitarian crises resulting from natural disasters (e.g., earthquakes, tsunamis, floods, and droughts); armed conflict; or civil strife routinely lead to large-scale population displacements. Whether migrating outside their countries as refugees or internally displaced in their homelands, disrupted populations routinely experience increased illness and death from respiratory and diarrheal pathogens associated with overcrowding; disrupted health services (e.g., childhood immunizations, treatment for HIV and tuberculosis); and lost access to food, clean water, and sanitation (*19**,**20*). For >50 years, CDC has provided technical support to WHO, United Nations agency partners, and others to define the public health aspects of such complex humanitarian emergencies and establish disease surveillance and interventions to mitigate the health consequences of displacement (*21**–**24*).

The number of persons affected by complex emergencies has increased over the past decade. In 2016 alone, >125 million persons needed humanitarian assistance (*25*). During 2007–2016, CDC responded to >20 crises that each affected >10,000 people, each with a crude mortality rate of >1/10,000 persons/day (e.g., the 2010 earthquake in Haiti, the Horn of Africa drought and famine of 2011–2014, and the Syrian crisis since 2012). During 2011–2016, CDC deployed staff for >380 missions in >40 countries to apply public health principles and epidemiologic science to mitigate the health impacts of complex emergencies (Table 1). For the crisis in Syria, CDC deployed staff who worked with nongovernmental organizations and the United Nations Children’s Fund to establish and train staff to conduct surveillance, measles vaccination campaigns, and nutritional surveys. In response to the 2011–2012 Horn of Africa famine, CDC worked with partners to implement morbidity and mortality surveillance systems in 3 countries (Table 1).

### Public Health Emergency Management Program

The terrorist attacks of September 11, 2001, intentional use of anthrax spores as a biologic agent during that same year, and increasing numbers of outbreaks and complex humanitarian responses prompted CDC to develop a US-based public health emergency management (PHEM) program (*26*). CDC initially implemented its incident management system (IMS) and activated its EOC in response to SARS outbreaks in 2002–2003. During 2001–2016, CDC has responded to 62 public health emergency events; on 244 other occasions, components of PHEM were used to support responses not requiring full EOC activation. In 2009, the PHEM program began assisting MOHs with strengthening their emergency preparedness through IMS trainings, EOCs, and emergency response plan development.

In 2013, in response to increasing global requests for more sustained emergency management training out of CDC’s EOC, CDC established its PHEM Fellowship Program. Also in 2013, CDC received accreditation from the Emergency Management Accreditation Program (*27*). CDC is now a recognized world leader in PHEM, providing technical assistance to GHSA and other countries (Figure 1).

### Global Disease Detection Operations Center 

CDC established the Global Disease Detection Operations Center (GDDOC) in early 2007 to identify and monitor health threats to the American public and global community. Using event-based surveillance for early alerting and situation awareness, a team of analysts routinely monitor numerous information sources (e.g., Internet, traditional and social media) for disease events using keywords in >50 languages, and validate accuracy with MOHs, WHO, Food and Agricultural Organization of the United Nations, World Organisation for Animal Health, and other partners. GDDOC monitors 30–40 public health threats daily, tracking situations that could develop into public health emergencies of international concern (*28*). During 2007–2016, GDDOC conducted event-based surveillance and disseminated information on >1,500 outbreaks occurring in >190 countries (Table 1).

GDDOC outbreak response support has included staff deployments and the provision of personal protective equipment, laboratory diagnostic test equipment, reagents, and supplies. GDDOC deploys CDC staff directly to the host country and through the WHO-hosted Global Outbreak Alert and Response Network. Emergency funding from the US Congress in 2014 for Ebola and GHSA made it possible to dramatically augment bilateral and Global Outbreak Alert and Response Network deployments for the West Africa Ebola epidemic. During 2016–2017, GHSA support substantively enhanced GDDOC’s capacity to conduct event-based surveillance.

### Global Disease Detection Regional Centers

**Figure 2:**
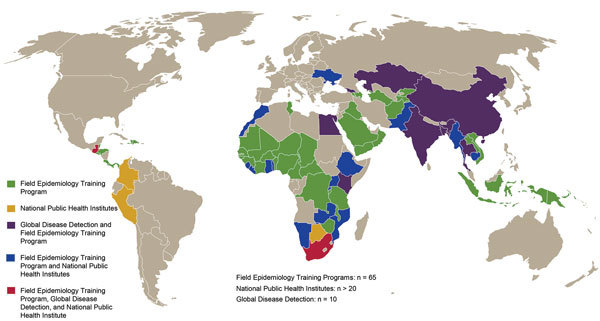
Selected programs that enhance US Centers for Disease Control and Prevention (CDC) global health protection platform. This map does not include CDC international influenza, malaria, HIV/AIDS, and immunization programs.

Following the 2002–2003 SARS outbreak, in 2004, the US Congress authorized funding for CDC to establish a regional Global Disease Detection (GDD) Program. Currently, CDC works with MOHs in 10 countries (Table 1; Figure 2) to host GDD Centers in a network supporting >90 countries. GDD Centers develop public health capacity by conducting epidemiology-, informatics-, and laboratory-based activities and scientific research. GDD Centers characterize public health threats through surveillance, applied research, and pathogen detection and discovery. During 2006–2016, GDD Centers conducted surveillance for key infectious diseases and syndromes; established >380 new diagnostic tests in national or local laboratories in 59 countries; assisted in the discovery and/or detection of 79 strains or pathogens new to the world, country, or region, responded to 2,051 requests for disease outbreak assistance; and trained 115,566 professionals at the national and regional level on public health topics (Table 1). Increasingly, GDD Centers are leading applied research and surveillance efforts to identify the most effective and efficient capacity-building activities that ensure health security.

## Capacity-Building Partnerships to Contain Threats at the Source

### Global Health Security Agenda Implementation

With growing recognition that infectious disease outbreaks can become pandemics, resulting in considerable loss of life and economic cost, GHSA was launched in February 2014 by 29 countries, WHO, the Food and Agricultural Organization of the United Nations, and the World Organisation for Animal Health to rapidly identify and mitigate infectious disease threats at their source (*15*). Now, >60 nations are GHSA member countries (Figure 1). The group of 7 industrialized democracies (G7), South Korea, Canada, Nordic countries, and a growing list of private partners have pledged financial support for GHSA implementation in up to 76 countries (Figure 1).

In 2015, the US government committed to accelerating GHSA implementation with 31 countries and the Caribbean Community, an organization of 15 island nations (Figure 1). The United States is investing >$1 billion to advance GHSA’s prevent, detect, and respond framework against infectious disease threats through implementation of 11 measurable action packages (Table 2) (*14**–**16*). In 17 Phase I countries, 14 Phase II countries, and the Caribbean Community (Figure 1; Table 1), CDC supports country capacity assessments, 5-year roadmaps, and annual GHSA implementation plan development. In addition, in Phase I countries, CDC provides financial support for implementation of these action packages; substantial progress was achieved in the first year (*16**)*. To reduce the risk of emergent zoonotic infections, 13 countries have expanded surveillance systems in humans, wildlife, and animals to foster prevention (Table 1). Ten countries have expanded surveillance systems to include more vaccine-preventable diseases (VPDs), which should strengthen national vaccine delivery systems, including the capacity for emergency vaccination to mitigate an outbreak. For example, community-level monitoring can accelerate targeted immunization, halving the number of vaccine-preventable meningococcal disease cases in West Africa outbreaks (*29**,**30*).

To enable disease detection and response efforts, a strong national reference laboratory system requires a tiered laboratory network, including capable central reference laboratories linked to regional and peripheral laboratories with appropriate testing capacities at each level; systems for timely and safe transport of samples and return of results; and procedures that assess and ensure quality. GHSA resources have supported enhanced training for laboratory technicians in 17 Phase I countries, and 16 countries have detected dangerous pathogens using new equipment (Table 1). All 17 Phase I countries have established or expanded the training of field-based epidemiologists, thereby greatly enhancing the number of staff that can detect and effectively respond to health threats at the subjurisdictional level.

A national IMS with coordination of response through EOCs is essential for mitigating public health threats. Sixteen Phase I countries have established or strengthened their national EOCs to manage and monitor health events in real time; of these, 11 have activated their EOCs for simulated and/or real emergency responses.

### Joint External Evaluation 

**Figure 3:**
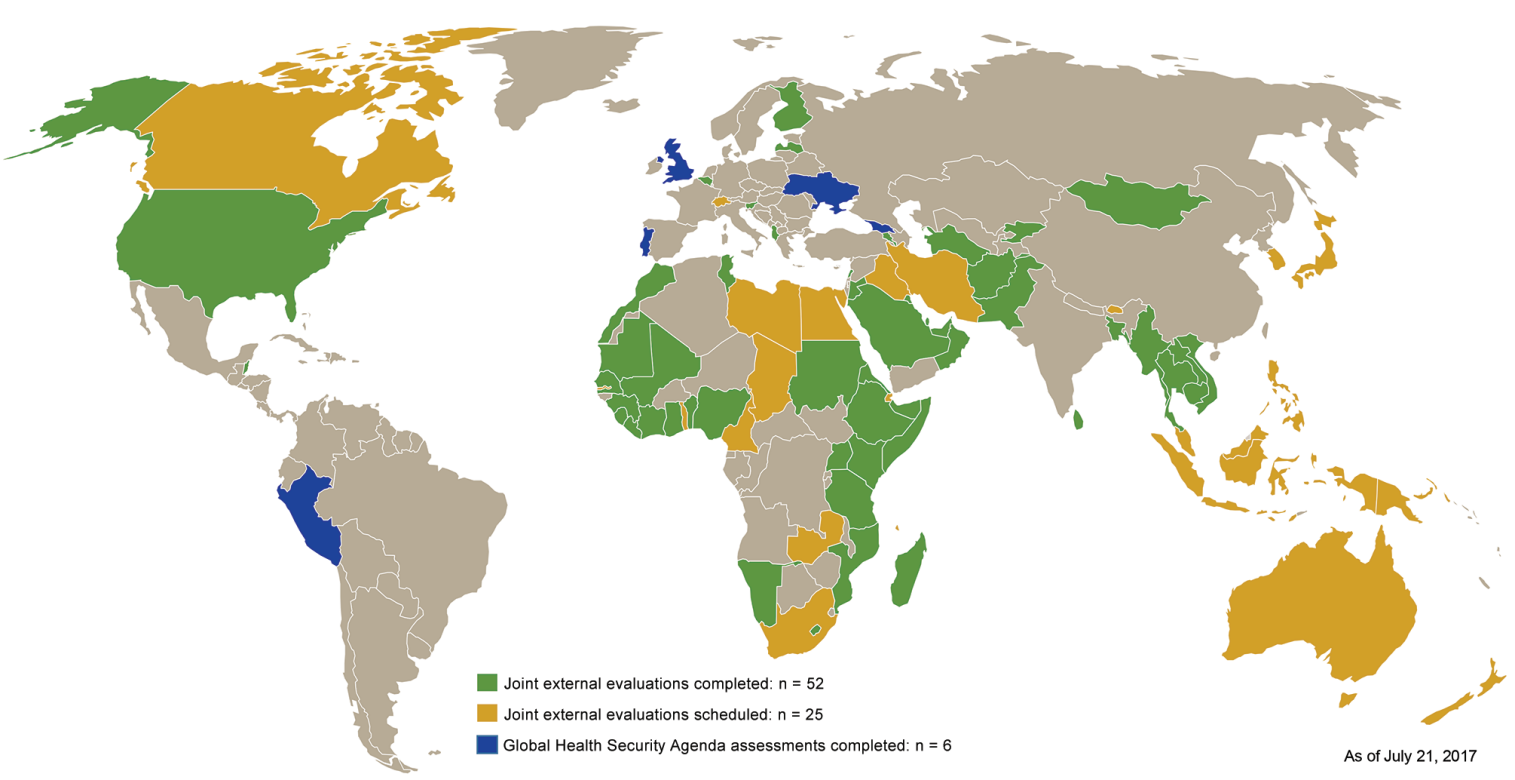
Country progress with independent Global Health Security Agenda and Joint External Evaluation assessments through 2018.

With so few countries meeting their IHR 2005 commitments through 2014, a validated monitoring program to measure and facilitate progress toward compliance was needed. With CDC support, external and independent GHSA assessments were piloted throughout 2015 in 6 countries to establish a baseline for targeting implementation (Table 1; Figure 3). In February 2016, WHO, working with CDC and GHSA partner countries, adopted the Joint External Evaluation (JEE) tool to harmonize independent monitoring for both GHSA targets and IHR 2005 compliance efforts across all 19 IHR core preparedness capacities (*31*). JEEs are designed to establish a baseline measurement for a country’s capacity, inform national policy setting, target resources, track progress, and highlight priority areas for improvement.

By mid-July 2017, 52 JEE country assessments were complete, and 27 JEE reports were publicly posted. An additional 25 countries are scheduled for a JEE through 2018 (Table 1; Figure 3).

### Public Health Workforce Development

A well-trained and retained public health workforce is a cornerstone for achieving IHR compliance. CDC is helping develop a global workforce through the FETP-Advanced, modeled after CDC’s 2-year Epidemic Intelligence Service (*11*). Since FETP’s inception outside North America in 1980, CDC has supported FETPs-Advanced in 65 countries and graduated >3,900 advanced field epidemiologists (Table 1; Figure 2); of these graduates, up to 80% continue to serve in public health programs in their home countries (*12*). In GHSA Phase I countries, >1,600 persons have completed FETP training. In 2001, CDC started an FETP-Intermediate of 6–9 months’ duration to address district-level public health surveillance and outbreak response gaps in 13 countries. Through 2016, >700 disease detectives had completed intermediate training. During 2005–2016, FETP-Advanced graduates conducted >3,300 outbreak investigations (Table 1). In response to the West Africa Ebola epidemic, FETP prioritized the expansion of FETP-Frontline programs, providing a 3-month training for district surveillance officers to improve local disease detection and response. During 2014–2016, CDC supported 24 new FETP-Frontline programs, mentoring >1,860 participants (Table 1). 

### International Influenza and Respiratory Diseases

Since the 1980s, CDC has supported influenza surveillance and laboratory capacity globally. As of June 2017, CDC supports influenza activities in 79 countries, assisting MOHs and other laboratory partners in the Global Influenza Surveillance and Response Network in the early detection of potential pandemic threats and provides the world with access to new influenza strains to enable the development of effective seasonal influenza vaccines and vaccines against novel influenza strains that have pandemic potential (e.g., the H7N9 avian strain currently circulating in China) (*32*). With GHSA support, the online International Reagent Resource portal (https://www.internationalreagentresource.org/) has provided reagents to national influenza laboratories and other respiratory disease laboratories worldwide.

CDC has supported global laboratory networks for polio, measles, and rubella for several decades, providing diagnostic testing, technical support, training, and reference laboratory services. Since 2001, CDC has worked with MOHs, WHO, and the Meningitis Vaccine Project, funded by the Bill and Melinda Gates Foundation, to develop and administer meningococcal vaccines to millions of persons living in the Africa meningitis belt and leads the MenAfriNet Consortium to enhance surveillance to monitor the effectiveness of vaccines and emergence of new meningococcal strains (*33*).

GHSA supports MOHs to conduct surveillance for severe respiratory diseases and other illness clusters. To support this effort, laboratories are provided with test kits and reagents, packaging and shipping protocols, and training in advanced molecular testing methods, allowing detection of multiple pathogens simultaneously.

### International Emerging and Zoonotic Diseases

Most known, new, or emerging infectious disease threats are zoonotic in origin (*34**,**35*). Zoonoses are responsible for an estimated >2 billion human illnesses and 2 million human deaths annually (*36*). Under GHSA, many countries are undertaking efforts to identify and prioritize zoonotic diseases of greatest national concern through a One Health approach (i.e., linking human, environmental, and animal health) (*37*). This approach helps a country focus limited resources for surveillance, laboratory capacity building, outbreak response, and prevention and control efforts and helps to enhance communication, collaboration, and engagement across critical sectors of government. With technical assistance from experts on zoonotic and emerging infectious diseases, many countries have initiated surveillance to establish etiologies of acute febrile illness. These efforts have begun to increase countries’ capacity to collect sterile specimens; prepare, store, and ship specimens; and collect and report data to clinicians and surveillance systems. Acute febrile illness surveillance has contributed to countries’ understanding of etiologies and pathogen-specific disease burden and can inform clinical algorithms and care and treatment of patients with acute febrile illness. GHSA implementation has demonstrated that enhancing disease-specific capacity improves national public health capacity building overall. Coordinated efforts between cholera experts and emergency management to prevent, detect, and respond to cholera in Cameroon have led to increasing timeliness of EOC activation for other outbreaks. Preventing zoonotic or emerging infectious diseases is one of the critical tenets of GHSA. CDC infection, prevention, and control experts are supporting efforts to build infection control and antimicrobial drug resistance capacity in 10 GHSA Phase I countries. During the West Africa Ebola epidemic, widespread gaps in infection, prevention, and control systems and resources led to outbreak amplification (*38*). Today, these national policies and practice guidelines are in the Ebola-affected countries to help support sustainability of these efforts.

### Global VPD Activities

Country capacity to conduct high-quality VPD surveillance is critical to increase coverage to prevent, detect, and respond to VPD outbreaks. CDC has supported global laboratory networks for polio, measles, and rubella for several decades, providing diagnostic testing, technical support, training, and reference laboratory services. An effective multidisciplinary workforce, including epidemiologists, laboratorians, and data managers, is needed to collect, analyze, and report VPD surveillance data that are accurate, timely, and useful for decision making.

Since 1998, CDC has provided technical and financial support to develop VPD surveillance capacity in low- and middle-income countries through the Stop Transmission of Polio (STOP) program of the Global Polio Eradication Initiative (*39*). Through July 2017, a total of 2,010 STOP volunteers have been trained in surveillance principles to detect and respond to polio and other VPDs. These volunteers have deployed in 48 teams for 3–6-month assignments to 77 countries (Table 1) (*39*). STOP volunteers have played a crucial role in enhancing country capacity to respond to outbreaks of other priority infectious diseases, contributing to CDC’s global health protection platform. CDC also has supported 4 countries at high risk for polio to build their own national STOP programs (Table 1) (*40**–**42*). VPD surveillance also helps build countries’ public health systems. For example, in Nigeria in 2014, the polio EOC quickly converted to respond to Ebola (*43*).

### National Public Health Institutes 

National governments are responsible for keeping their citizens healthy and addressing public health challenges. To that end, many countries have established national public health institutes (NPHIs) to carry out essential public health functions, including outbreak detection and response (*44**,**45*), and facilitate progress toward IHR 2005 compliance. CDC is the US government’s NPHI and is 1 of >100 members representing 88 countries in the International Association of National Public Health Institutes (IANPHI) (Table 1). With IANPHI, CDC directly supports >20 IANPHI countries in establishing or strengthening their own NPHIs (Figure 2) through developing strategic plans aligned with public health priorities, determining necessary policy changes, creating sustainability plans, and providing technical assistance (Table 1).

### Public Health Implications and Future Directions

Outbreaks, regional epidemics, and pandemics are costly (*46**–**50*). During February–July 2003, SARS spread across 4 continents, infected 8,100 persons, killed 774 persons, and cost the global economy $40 billion (*46*). In the first year of the 2009 influenza H1N1 pandemic, >575,400 persons succumbed worldwide (*47*). A severe influenza pandemic could cost as much as 4.9% of the world’s gross domestic product (*48*). In 2015, the West Africa Ebola epidemic cost Guinea, Liberia, and Sierra Leone about $2.2 billion (*49*).

Because of the recognized need to achieve IHR 2005 compliance worldwide to ensure health security, increasing number of countries that have made GHSA commitments, and early progress achieved with GHSA implementation, the world is becoming better prepared to respond to threats. CDC is helping advance health security through its global health protection platform. More work is needed and momentum in GHSA implementation needs to be sustained so Americans and citizens around the world will have enhanced protection from newly emerging infectious diseases and other health threats.
